# Integrating Functional and Diffusion Magnetic Resonance Imaging for Analysis of Structure-Function Relationship in the Human Language Network

**DOI:** 10.1371/journal.pone.0006660

**Published:** 2009-08-17

**Authors:** Victoria L. Morgan, Arabinda Mishra, Allen T. Newton, John C. Gore, Zhaohua Ding

**Affiliations:** 1 Vanderbilt University Institute of Imaging Science, Nashville, Tennessee, United States of America; 2 Department of Biomedical Engineering, Vanderbilt University, Nashville, Tennessee, United States of America; 3 Department of Electrical Engineering and Computer Science, Vanderbilt University, Nashville, Tennessee, United States of America; 4 Chemical and Physical Biology Program, Vanderbilt University, Nashville, Tennessee, United States of America; University of Regensburg, Germany

## Abstract

**Background:**

The capabilities of magnetic resonance imaging (MRI) to measure structural and functional connectivity in the human brain have motivated growing interest in characterizing the relationship between these measures in the distributed neural networks of the brain. In this study, we attempted an integration of structural and functional analyses of the human language circuits, including Wernicke's (WA), Broca's (BA) and supplementary motor area (SMA), using a combination of blood oxygen level dependent (BOLD) and diffusion tensor MRI.

**Methodology/Principal Findings:**

Functional connectivity was measured by low frequency inter-regional correlations of BOLD MRI signals acquired in a resting steady-state, and structural connectivity was measured by using adaptive fiber tracking with diffusion tensor MRI data. The results showed that different language pathways exhibited different structural and functional connectivity, indicating varying levels of inter-dependence in processing across regions. Along the path between BA and SMA, the fibers tracked generally formed a single bundle and the mean radius of the bundle was positively correlated with functional connectivity. However, fractional anisotropy was found not to be correlated with functional connectivity along paths connecting either BA and SMA or BA and WA.

**Conclusions/Significance:**

These findings suggest that structure-function relations in the human language circuits may involve a number of confounding factors that need to be addressed. Nevertheless, the insights gained from this work offers a useful guidance for continued studies that may provide a non-invasive means to evaluate brain network integrity *in vivo* for use in diagnosing and determining disease progression and recovery.

## Introduction

Understanding structure-function relations of the human brain is a fundamental goal of neuroscience research. In particular, a knowledge of structural connectivity between functionally connected cortical regions provides us insights regarding the architecture of the distributed neural networks in the human brain [1]. Characterization of the structural connectivity between brain cortical regions has now become feasible with the advent of a novel MRI technique called diffusion tensor imaging (DTI). DTI provides, for each voxel, a tensor matrix that describes the constraints on local Brownian motion of water molecules. Originally proposed to assess tissue properties such as diffusion anisotropy [2], [3], DTI has recently evolved to become the primary modality for mapping neuronal fiber tracts in vivo, a technique widely recognized as DTI tractography. DTI tractography draws upon the principle that the dominant direction of water diffusion (principal diffusion direction) coincides with the local tangent direction of fibrous tissue, and integration of the principal diffusion directions permits entire fiber tracts to be delineated [4], [5]. Based on these, a number of streamline-like fiber tracking algorithms have been developed to generate the pathways of fiber connections [6]–[10]. To account for uncertainties associated with the principal diffusion direction, probabilistic fiber tracking methods which provide probability maps of fiber connectivity are also proposed [11]–[15]. This structural connectivity has been found to be correlated with functional ability across several networks in the brain [16]–[19].

In addition to structural connectivity, MRI can be used to measure functional relationships between brain regions using blood oxygen level dependent (BOLD) [20]–[22] functional MRI (fMRI). BOLD fMRI is a technique that creates images in which the signal intensity depends on the amount of deoxyhemoglobin in the blood within the tissue. The amount of deoxyhemoglobin is modulated locally by changes in neuronal activity, blood flow and oxygen consumption and is, therefore, used as a marker of brain activity. In the steady-state, near infrared spectroscopy has been used to show spontaneous, low frequency oscillations of these hemodynamic effects [23], [24]. Biswal et al. first demonstrated that low frequency oscillations of BOLD signals measured with MRI are linearly correlated between regions of the motor system across the brain [25]. Since then, correlations in low frequency BOLD oscillations have been found across many networks in the brain including motor [26]–[28], language [29]–[31], and vision [32] pathways (see reviews [33], [34]). In fact, the measurement of functional coupling between brain regions using correlations in low frequency fMRI BOLD oscillations reveals functional connectivity between these regions. Like structural connectivity, changes in functional connectivity have been reported in disease states and healthy controls correlated with reduced functional ability [35]–[38] and correlated with cognitive abilities [39]. However, to date there have been a limited number of studies that relate these different approaches or try to correlate functional and structural connectivities. Indeed, they measure quite different features of the brain so it is still unclear to what degree they may be related.

In order to examine this relationship, some investigators have independently analyzed DTI and fMRI data acquired in the same session [40], [41], or in separate subjects [42], attempting to characterize both structural connectivity using DTI and location of fMRI activity in healthy and pathological conditions. The combination of DTI and fMRI measures into a single analysis may provide unique information not available with either single modality [1]. Most commonly the functional information is used to guide the fiber-tracking by defining functional regions [43]–[53] or the magnitude of activity is compared to the magnitude of structural connectivity [50], [51], [54]–[56].

Although these studies have implemented both DTI and fMRI measures of activity to study a single network or stimulus, only a few reports were found that directly related fMRI functional connectivity and DTI derived structural connectivity in a single network. In one study these measurements were limited to two adjacent gyri in the frontal lobe [11] with results that showed that the relationship between these two measures is complex. High functional connectivity was found between regions with low structural connectivity, possibly due to fibers not contained within the imaging slice or indirect structural connections; but low functional connectivity was not found between regions with high structural connectivity. In a second study involving patients with multiple sclerosis, structural connectivity measured as FA was found to be positively correlated with functional connectivity only when the controls and patients were combined [57]. Skudlarski et al. [58] looked at functional vs. anatomic connectivity across the whole brain and in anatomically defined regions and found good overall spatial agreement between the two types of connectivity maps. They also found that agreement between the individual measures is increased when the individual measures themselves are increased.

In this study, our goals were to develop techniques for integrated structure and function studies of the human brain, and to apply the techniques developed to examine and quantify the relationship between MRI-derived structural and functional connectivity between more distal intra-hemispheric regions in healthy controls using whole brain MRI acquisitions. As an initial attempt, we focused on the human language network, in which the connection patterns have been reasonably well established. Specifically, DTI structural connectivity indices were compared to fMRI functional connectivity indices between regions activated in a series of language tasks in the left frontal (premotor and Broca's area) and the left parietal temporal region (Wernicke's area) in a population of right-handed, healthy controls. We hypothesize that this analysis will more directly elucidate any linear relationships existing between MRI structural and functional connectivity between functionally activated regions across a network.

## Materials and Methods

### Ethics Statement

This study was approved by the Vanderbilt University Institutional Review Board. Written informed consent was obtained prior to MRI scanning each subject in accordance with the Vanderbilt University Institutional Review Board guidelines and the ethical standards laid down in the 1964 declaration of Helsinki.

### Imaging

Twelve normal healthy volunteers (6M, 6F, mean age±stdev: 27.8±5 years) participated in this study. All were right handed by self-report. No subjects had a history of neurological, psychiatric or medical conditions as determined by interview.

All imaging was performed on a 3T MRI scanner (Philips Medical Systems, Best, Netherlands) with a 8-channel head coil. Subject motion was minimized using pads placed within the head coil. All stimuli were programmed using Matlab with the Psychophysics toolbox (The Mathworks, Natick, MA). Visual display was provided by MRI-compatible backprojection (Avotec Inc., Stuart, FL) onto a small screen behind the head coil. Headphones within the MRI system provided auditory stimuli.

Structural imaging included a three-dimensional high-resolution T1-weighted volume (1 mm×1 mm×1 mm) and a two-dimensional high-resolution T1-weighted image set in the same slice locations as the functional scanning (0.94 mm×0.94 mm×5 mm). Functional BOLD scanning was performed using a T2*-weighted gradient echo, echo planar imaging sequence (64×64, FOV = 240 mm, TE = 35 ms, TR = 2 sec, 4.5 mm thickness, 0.5 mm gap, 30 slices, 100 volumes). Four functional tasks were performed by each subject to localize regions of the fronto-temporal language network – *word generation from categories*, *word generation from letters*, *reading*, and *listening* – in a block design format with ten 20 second blocks starting with control. In *word generation from categories* the subject was visually presented one category name in a single block (i.e. food) and was instructed to silently think of words that fit in the given category [59]. The control was a black box on a white background. In *word generation from letters* the subject was visually presented a single letter in each block and was instructed to silently think of words that begin with that letter [60], [61]. The control for this task was also a black box on a white background. For the *reading* task, the subject was visually presented small phrases describing a noun (i.e. a long yellow fruit) every four seconds during the block [62]. This was contrasted with strings of symbols. The subject was instructed to read the phrases and look at the symbols. The *listening* task involved auditory presentation of a person reading a story contrasted with the same block of auditory stimuli presented in reverse. The subject was instructed to close their eyes and listen carefully. A fifth fMRI BOLD scan was acquired for the functional connectivity analysis with the same parameters except for 200 volumes (6∶40 scan time) with the subject instructed to close eyes and relax.

Diffusion weighted images were acquired using a single shot, echo-planar, pulsed gradient spin echo imaging sequence with a *b* value of 1000 s/mm^2^ along 32 non-collinear weighting directions (128×128, FOV = 256 mm, TE = 60 ms, TR = 10 sec, 2 mm thickness, 0 mm gap). To improve the signal-to-noise ratio of the images, three repeated scans were obtained, which were subsequently co-registered and averaged using DTI processing utilities in the Philips PRIDE workstation. Tensor elements were then calculated from the averaged diffusion weighted images. Finally, tensor characteristic parameters including the principal diffusion direction (PDD) and fractional anisotropy (FA) were derived from the tensor elements.

### Region of Interest Identification

Group activation maps were calculated and used to localize three functionally activated language regions across subjects and then the individual subject's activation within the group regions was used for both connectivity analyses. The three language regions identified were Broca's area in the left inferior frontal gyrus (BA 44/45), the superior medial frontal gyri in the supplementary motor area (BA 6/8), and Wernicke's area in the left superior temporal gyrus (BA 22).

The images were analyzed using the following procedure using SPM5 [http://www.fil.ion.ucl.ac.uk/spm/software/spm5/]. All functional BOLD image sets were corrected for slice timing artifact, motion corrected and spatially normalized to the MNI template [63] using co-registration to the three-dimensional and two-dimensional T1-weighted structural image sets as intermediate steps and interpolated to 4×4×4 mm voxels. The *word generation from categories* and *word generation from letters* BOLD images were analyzed together using a fixed-effects general linear model on an individual subject basis to determine activation including Broca's area and the supplementary motor area for each subject (map1). The *reading* and *listening* tasks were analyzed in the same way to localize activation including Wernicke's area for each subject (map2).

**Figure 1:**
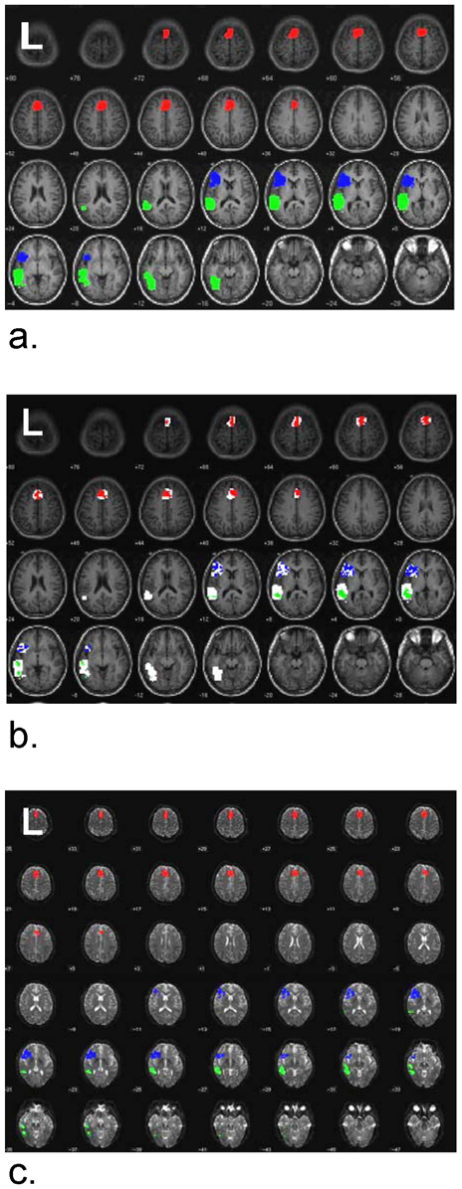
Group and individual regions of interest. a) Three different regions of interest determined using group activation maps at *t*>3 cluster size 10 identified in MNI template space with voxel size 4×4×4 mm^3^; b) The same region of interest masks shown in white with a single subject's regions used for functional connectivity analysis shown within; c) The same subject's regions of interest transformed into individual DTI image space used for DTI tractography with voxel size 2×2×2 mm^3^. Red = supplementary motor region (SMA), blue = Broca's area (BA), and green = Wernicke's area (WA).

To determine group activation, all of the individual subjects' contrast maps of map1 were entered in a one-sample t-test using SPM5. Activation at the threshold of *t*>3 with a cluster size of 10 (approximately *p*<0.001 corrected for multiple comparisons based on AlphaSim software [64]) was arbitrarily chosen to generally determine group activation regions. The regions were then smoothed using a 6 mm FWHM kernel. Regions of interest in Broca's area and supplementary motor area were identified on the group map and these two regions were used as the Broca's region and supplementary motor region group masks. The same method was used with the subjects' map2 to determine a Wernicke's area group mask (see Figure 1a).

Next, the individual subject's map1 activation thresholded at *t*>3 cluster size 10 that was contained within the Broca's area group mask was saved as the individual's Broca's area region of interest (BA). The same procedure was used to define the individual's supplementary motor region of interest (SMA) and Wernicke's area (WA) region of interest. These regions were determined for each individual subject (Figure 1b).

In order to perform the DTI tractography analyses, the three regions of interest for each subject had to be transformed from the MNI template space to the individual's DTI image space. This was done by spatially normalizing the MNI template to the individual's DTI T2* (b0-image)-weighted image set and then applying this transformation to the regions of interest on a subject by subject basis (Figure 1c). Although this second transformation may induce minor registration and smoothing error, it allowed the identification of voxels in the native space for DTI analysis. We expect that error will be smaller than that caused by the further manipulation required to apply spatial transformations to diffusion tensors [65], [66].

### fMRI Functional Connectivity Analyses

The pre-processed and spatially normalized functional BOLD images acquired at rest with eyes closed were used for fMRI connectivity analyses. For each subject, a global time course was determined by averaging across all voxels in the brain. This global time course, as well as the six motion time courses from SPM5, were regressed out of all of the voxels in the brain. Next, all voxels were low pass filtered with a cutoff frequency of 0.1 Hz. Applying the three regions of interest identified above to the individual's resting-state data, the average time course in the source region was determined and its linear correlation with every other voxel in the brain was calculated. Then the average correlation coefficient in the target region was determined. The correlation coefficient was transformed to a Z-statistic using the Fisher's Z-Transform. This value was considered an index of functional connectivity (FC) between the source region and the target region which could be compared across subjects. These calculations were performed for two pairs of regions: path 1 = BA (source) to SMA (target) and, path 2 = BA (source) to WA (target).

### DTI Structural Connectivity Analysis

#### Adaptive fiber tractography

The cortical regions involved in language processing usually resides in the gray matter, where the diffusion of water molecules is more isotropic in nature, due to which the FA (fractional anisotropy) is low in this region. The adjoining regions of white and gray matter interface are also less anisotropic and the accurate estimation of tensor element vectors is largely influenced by noise. At this point we hypothesize that each point on a structural pathway between functionally active regions has an uncertainty associated with tracking, which is a function of noise, partial volume effects and the degree of anisotropy of water molecular diffusion as well. The principal diffusion direction (PDD) becomes a less reliable indicator of the true fiber directions in the adjoining area, hence the conventional tracking methods may not provide the true fiber pathways. In this study, an adaptive fiber tracking approach is implemented for this purpose. The algorithm is similar to that proposed by Hagmann et al. [13], but improves upon it by allowing adaptive modulation of the tracking direction so that it behaves more like a deterministic fiber tracking in the white matter region but is of more probabilistic nature in the gray matter region. A clear advantage of the adaptive fiber tracking is that the tracking efficiency may be greatly enhanced since much fewer repeated trials are needed. Specifically, instead of simply following the PDD at each tracking step, the tracking direction is perturbed by a random vector that is added to the PDD; the degree of the random perturbation is modulated by local FA value. Equations 1–3 below govern the adaptive fiber tracking process:
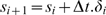
(1)

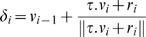
(2)

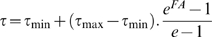
(3)


In the above equations, *s_i_*, *s_i+1_* are tract positions at step i, i+1 respectively, *Δt* is the tracking step size, and 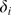 is the tracking direction for step i. The tracking direction 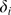 is determined by the PDDs of the current step (*ν_i_*) and previous step (*ν_i-1_*, providing tracking inertia), and a unit random vector *r_i_*. The relative contributions of *ν_i_* and *r_i_* are regulated by the parameter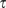, which is a sigmoid function of FA. The boundary values 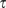
_min_ and 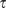
_max_ are respectively 0.3 and 3.0 in this study, selected empirically on the basis of trials.

According to this algorithm, in the white matter where the FA is high and the PDD is a more reliable indicator of the true fiber direction, the random perturbation to the tracking direction will be small, thus allowing the fiber tract to follow the local PDD more faithfully; in the gray matter where the FA is low and the PDD tends to be an unreliable indicator of the true fiber direction, the random perturbation will be large, thereby permitting the tracking direction to deviate significantly from the local PDD. When a large number of trials each with a different realization of random perturbation is performed, a fraction of fiber tracts will be able to reach the functionally active regions in the gray matter.

#### Fiber tracking procedure

**Figure 2:**
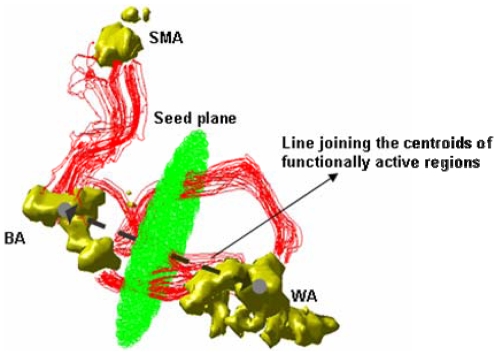
Diagram of DTI tracking procedure. Centroids of BA and WA are shown as gray spheres connected by a dashed black line. The seed plane used for origin of path 2 (BA to WA) normal to the line joining the centroids is shown in green. Examples of tracts connecting regions of interest are illustrated in red.

To initiate the adaptive fiber tracking, two circular seed planes were first defined such that they were respectively midway at and perpendicular to the line joining the centroids of BA and SMA of path 1, and that connecting the centroids of BA and WA of path 2 (Figure 2). The seed planes were sufficiently large to intersect all possible fiber pathways connecting two target regions of interest (ROI). A total number of 8000 iterations of fiber tracking were launched from 2000 uniformly sampled seed points in each of the planes (4 per voxel). Fiber tracking proceeded in both directions from the seed planes according to Equations 1–3 above, and was terminated when it reached a point with FA<0.1, or when it reached an ROI. Fibers that connected both target ROIs were stored for further analysis. We note that the purpose of using a small FA value as a termination criterion for the tracking process was to prevent it from entering the regions containing cerebral-spinal fluid, but still permitting it to traverse white matter and gray matter regions.

#### Structural connectivity measures

The above fiber tracking process yields fiber pathways that connect a pair of ROIs. In the white matter region, these pathways are still largely streamlines since random perturbations to them are pretty small due to high FA in the white matter (see Figure 2). Hence, characterization of structural connectivity between a pair of ROIs was based on these streamlines in this study. From the streamlines, two measurements were made that served as indices of structural connectivity between a pair of ROIs. The first was the mean FA of all unique voxels in the connecting tracts within a mask containing only the white matter. The white matter mask was obtained by using the segmentation utility in SPM5 (http://www.fil.ion.ucl.ac.uk/spm/software/spm5) to segment the white matter in the native space of each individual subject. The second structural connectivity measurement was made by analyzing the seed plane created between the pairs of ROIs. The center of all fiber crossing points on the seed plane between the ROIs was determined as the average x-coordinate and the average y-coordinate of those points. The mean distance (in voxels) of each fiber point on the seed plane to this center point was then calculated as the mean radius of the fiber bundle. Points on the seed plane where multiple unique fibers crossed were counted for the number of crossings in this calculation. This index of structural connectivity (mean radius) reflects thickness of a single fiber bundle between two ROIs. Calculation of mean radius in cases of multiple fiber bundles will be explained in the Results section.

### Structure and Function Comparisons

First, we compared the three measurements (FC, mean FA, and mean radius) between path 1 and path 2 using a paired t-test using SPSS 15.0 statistical software (SPSS, Inc., Chicago, IL). Next, we compared each of the two DTI-derived structural connectivity measurements with the FC index across both paths to identify any linear structure-function relationships. As path 1 was found to generally contain a single bundle, and therefore be more easily interpretable than path 2, the structural and functional connectivity measurements of this path alone were compared with linear correlation in a similar manner.

## Results

Activation maps were determined for both pairs of tasks in eleven of the twelve subjects. The subject without activation at the chosen level within the masked regions was excluded from further analyses. Among the eleven subjects whose functional activations were successfully detected, we were able to track fibers along path 1 between BA and SMA and those along path 2 between BA and WA for all cases except one, in which we failed to track fibers along path 2. Therefore, all further results will reflect those from the remaining 10 subjects.

**Figure 3:**
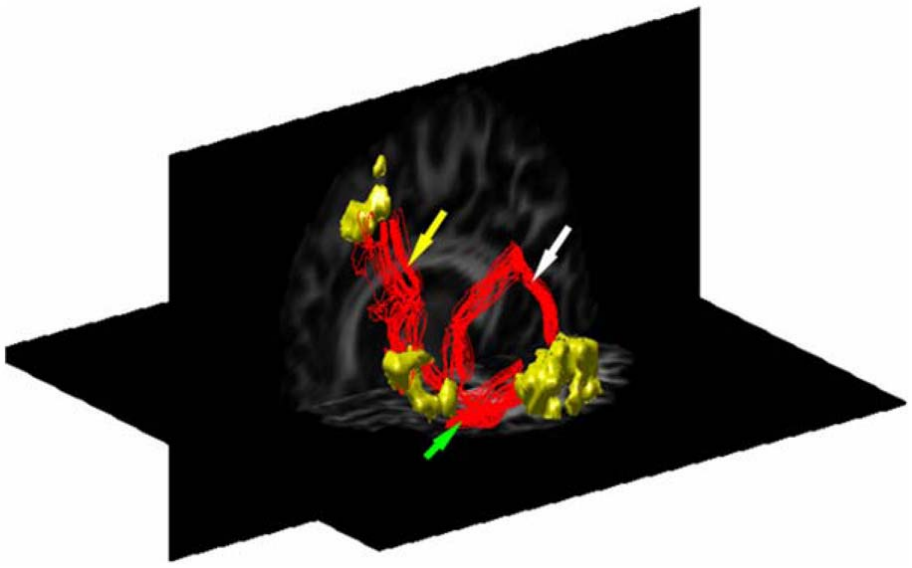
DTI tractography results in one subject. Connecting fibers are shown in the left hemisphere along path 1 (BA to SMA) (yellow arrow), and path 2 (BA to WA). In this case, path 2 fibers have two separable routes of connections – the dorsal route (white arrow) and ventral route (green arrow).

As an example, Figure 3 shows a 3-D view of path 1 (pointed by a yellow arrow) and path 2 in the left hemisphere overlaid on the FA map. Note that, in this case, path 2 fibers have multiple separable routes of connections – the dorsal route (pointed by a white arrow) and ventral route (pointed by a green arrow).

Among the ten subjects studied, all showed a dorsal route but only four of them had a ventral route and possibly a third connection route. These additional routes were not clearly definable. To measure the mean radius consistently, it was calculated only for the dorsal route for all the ten subjects.

When comparing the three measurements (FC, mean FA, and mean radius) between paths, the paired t-tests of the measurements between the paths showed that FC and mean radius were statistically different (mean path 1 vs. mean path 2; FC: 0.36 vs. 0.18, *p* = 0.005; mean radius: 3.39 vs. 5.89, *p* = 0.001), but difference in the mean FA between the paths did not reach a significance level (mean FA of path 1 = 0.39, mean FA of path 2 = 0.42, *p* = 0.025). This conclusion was based on the Bonferroni threshold (*p* = 0.05/3 = 0.017) for controlling a family wise error rate at 0.05.

**Figure 4:**
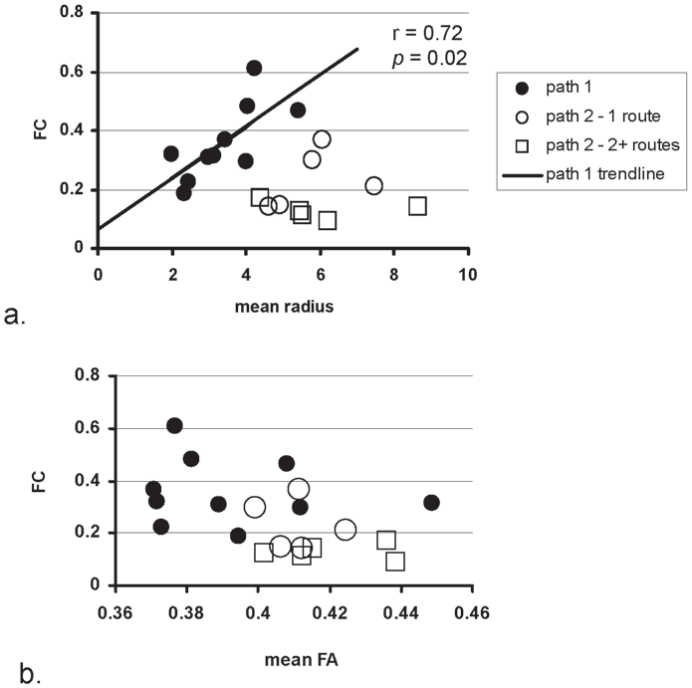
Structural vs. functional MRI measurements across two paths in the language network of healthy volunteers. Path 1 is between BA and SMA (solid symbols). Path 2 is between BA and WA (open symbols). Subjects with one or two routes of connection found between BA and WA are shown separately (1 route = circles, 2+routes = squares). FC is a measure of functional connectivity, while mean radius (a) and mean FA (b) are indices of structural connectivity.

The linear correlation analysis between the DTI-derived structural connectivity and the fMRI-derived functional connectivity yielded only one statistically significant relationship. For path 1, a positive linear relationship was found between FC and mean radius (r = 0.72, *p* = 0.02) (Figure 4a). The FC was not correlated with mean FA across either path in all subjects (*p*>0.3) suggesting no linear relationship between the two measures (see Figure 4b). Subjects with one or more than one route of connections between BA and WA of path 2 are shown separately.

## Discussion

This study is one of the first attempts to directly relate DTI tractography measurements with BOLD functional connectivity measurements in distal intra-hemispheric regions across the brain. We chose to focus this investigation on the language pathways that have been well studied and characterized with MRI including both fMRI activation, functional connectivity and DTI tractography. The combination of two separate paths between different regions of interest allowed for variation in the DTI and FC measurements to reveal some interesting relationships.

One main finding of this study was that FC increased linearly with increased mean radius along path 1. This tends to suggest the functional connectivity is supported by the structural connectivity along this path. However, FC was found not to be correlated with the mean FA along the path connecting either BA and SMA or BA and WA in the healthy controls. This seems to be inconsistent with the findings of Lowe et al. [57], who reported increased mean FA with increased FC. However, we propose that there are differences between the two studies which may account for this discrepancy. Firstly, the fiber tracts examined in that study were transcallosal motor pathways, but in the current study were the language pathways. Secondly, the previous study included patients whose data was required for the linear increase to be detected. Although they found no significant difference between the patients and controls in FC or mean FA, the mean FA from the controls appeared to be much more limited in range than the patients. In addition, the fiber tracking in the study by Lowe et al. was probabilistic, while in our study is more deterministic in the white matter where mean FA is computed.

In this work, we have found one connection route for path 1 but multiple connection routes for path 2 in a few of the subjects studied. This difference in general structure across the two paths and the use of the adaptive tracking method may explain why path 1 had significantly increased FC over path 2, but lower mean FA (although the difference did not reach a significant level after Bonferroni correction). This result infers that path 1 is a more direct connection consisting of more fibers that are more densely arranged in a smaller mean radius yielding higher FC. Path 2 may contain multiple connections of lower numbers of fibers arranged in narrower bundles over a larger cross section of the seed plane yielding lower total FC. The individual, narrower bundles of path 2 were tracked successfully using the adaptive tracking method because of their increased mean FA, whereas the lower FA of path 1 was successfully tracked with this algorithm because of its thicker radius allowing for continuation along the tract when the unit random vector was larger. These observations would lead to the conclusion that the seemingly inverse relationship between FC and mean FA across these two paths is not a direct one and that this finding is more a secondary effect of the differences in the general architecture of the two paths.

The finding of multiple paths between BA and WA has been reported elsewhere. Modern electrophysiological and tracer injection studies have recently suggested two separate routes connecting the auditory cortex to prefrontal areas [67], [68]: a dorsal route and a ventral route [69], [70], which perhaps correspond to different functions in language processing [71]. An earlier DTI study by Parker et al. [72] indeed confirmed the existence of two separate routes connecting the Broca's area and Wernicke's area. Hence, more detailed studies with structural connectivity along separate connecting routes and functional connectivity between segmented language regions may yield better insights regarding the structure-function relation in this path.

It might be that more linear structural/functional relationships exist in this network, but were not found in this study simply due to a number of technical limitations as follows. First, there are certain limitations in current DTI based fiber tractography. Most notably, the voxel size of the DTI data used in this study is 2×2×2 mm^3^, a few orders of magnitude greater than the size of individual axonal fibers. With this level of precision, which is nearly the best available from clinical scanners within a practical acquisition time, the PDD represents an averaged direction of a large number of individual fibers. This is known to affect the accuracy of the derived fiber tracts. Furthermore, the difficulty of tracking fibers and quantifying the structural connectivity between gray matter regions is widely recognized. While probabilistic or adaptive fiber tractography offers a viable solution for such a situation, to date it is still not fully certain that it yields a true picture of structural connectivity between gray matter regions, particularly in a quantitative manner. Second, our model of structural connections in the language circuits is based upon a canonical model that was established with primate electrophysiological and tracer injection studies [67], [68] There might be other undiscovered connections in humans that contribute to functional connectivity among the language cortices but were not included in our study of structural connectivity. For example, a DTI study by Catani et al. [73] suggested that, in addition to the dorsal and ventral routes, there may be indirect pathways connecting the Broca's and Wernicke's areas through inferior parietal cortex. Third, the low frequency correlation of steady-state fMRI signals between cortical regions, commonly used as a measure of functional connectivity, may not arise solely from axonal connections. These signals may also contain artifacts due to physiological sources such as respiration and cardiac pulsation [74], [75]. Also, the activated language regions identified in this study, with which both functional and structural connectivities were quantified, may vary with variable levels of effort and performance by individual subjects. Finally, inaccuracies in the experimental procedures such as imperfect co-registration may affect to some extent a combined study of functional and structural connectivity.

We have made one of the first efforts to investigate the structure-function relations in the human language circuitry using a combination of functional and diffusion MRI. When comparing the single bundle connection between BA and SMA, a positive correlation was found between the functional connectivity and the radius of the fiber bundle connecting the two regions. However, comparisons of functional connectivity with fractional anisotropy along paths connecting either BA and SMA or BA and WA yielded no significant correlations. These findings suggest that structural and functional connectivity in these language circuits may be affected by many more factors than are addressed here and that their relationship may be non-linear or weak. However, the insights gained from this work offers a useful guidance for continued studies that may provide a non-invasive means to evaluate brain network integrity *in vivo* for use in diagnosing and determining disease progression and recovery.
